# Acute aortic thrombosis in the ascending aorta after cisplatin-based chemotherapy for esophageal cancer: a case report

**DOI:** 10.1186/s40792-022-01431-8

**Published:** 2022-04-24

**Authors:** Noriaki Sato, Takehito Mishima, Yuka Okubo, Takeshi Okamoto, Shuichi Shiraishi, Masanori Tsuchida

**Affiliations:** Division of Thoracic and Cardiovascular Surgery, Niigata University Graduate School of Medical and Dental Sciences, 1-757, Asahimachi-dori, Niigata, Japan

**Keywords:** Acute aortic thrombosis, Chemotherapy, Thrombectomy

## Abstract

**Background:**

The risk of thrombus development is considered to be increased by malignant tumors and chemotherapy. In addition, thrombosis of the ascending aorta is rare. We report a case of ascending aortic thrombectomy in a patient with esophageal cancer who developed ascending aortic thrombus after starting neoadjuvant chemotherapy, including operative findings and surgical treatment.

**Case presentation:**

A 63-year-old man with esophageal cancer was administered chemotherapy comprising cisplatin plus 5-fluorouracil. A week after completing 1 cycle of chemotherapy, computed tomography angiography showed acute aortic thrombosis at the ascending aorta. The risk of embolization appeared high because the thrombosis was floating, so we performed emergency ascending aortic thrombectomy. The postoperative course was good and uncomplicated. A month after this surgery, the patient underwent surgery for esophageal cancer. As of 1 year after the cancer surgery, neither cancer nor thrombosis has recurred.

**Conclusion:**

We describe a case of acute aortic thrombosis in the ascending aorta after cisplatin-based chemotherapy, that was treated by aortic thrombectomy. The treatment strategy should depend on thrombus location and the condition of the patient, but surgical treatment should be considered where possible to achieve better prognosis.

## Background

The risk of thrombus development is considered to be increased by malignant tumors and chemotherapy [1]. Venous thrombosis is more common, but arterial thrombosis associated with cisplatin has been reported in a few cases. In addition, thrombosis was originally thought to occur mostly in the abdominal aorta to lower extremity arteries, but a few reports in recent years have described thrombosis in the ascending aorta. We report a case of ascending aortic thrombectomy in a patient with esophageal cancer who developed ascending aortic thrombus after starting neoadjuvant chemotherapy, including operative findings and surgical treatment.

## Case presentation

**Fig. 1:**
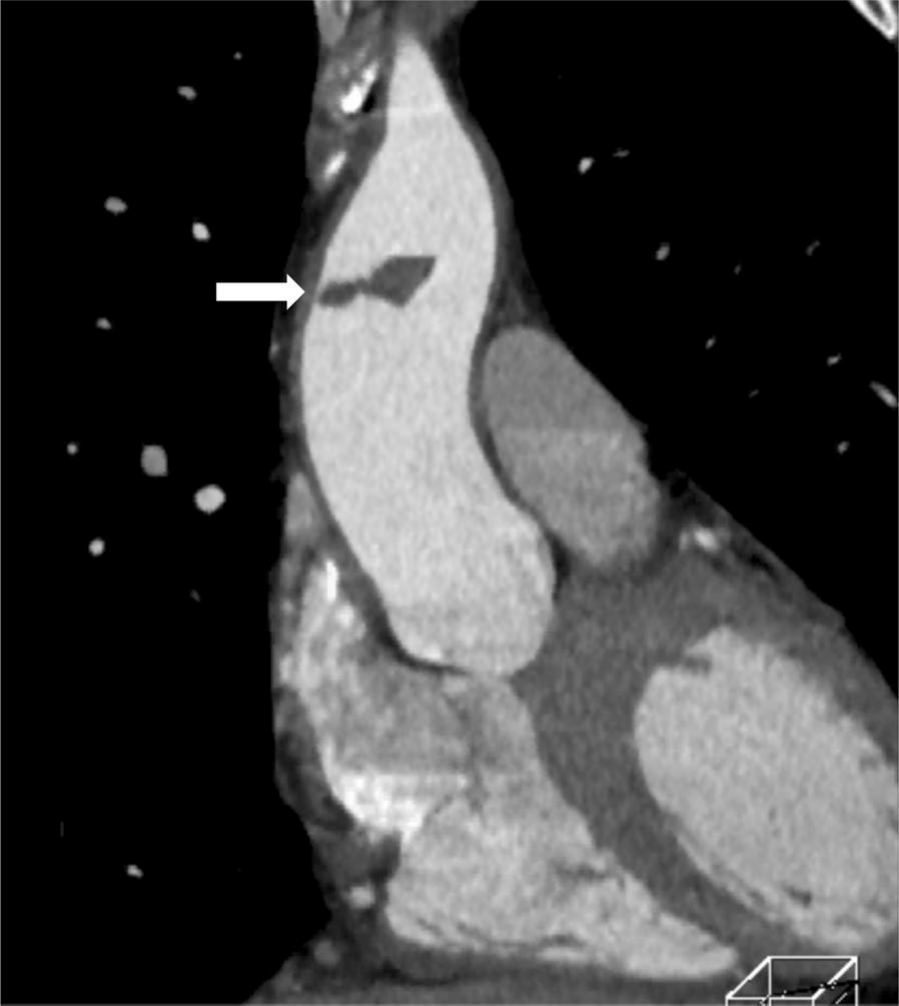
Computed tomography (CT) shows floating thrombosis (white arrow)

Physical examination of a 63-year-old man led to suspicion of gastric cancer. He had a history of hypertension and bronchial asthma. On referral to our hospital, esophageal cancer was also diagnosed in addition to the gastric cancer. Endoscopic submucosal dissection was performed for the early-stage gastric cancer. Neoadjuvant chemotherapy followed by surgical treatment was planned for the esophageal cancer. Chemotherapy was initiated with 5-fluorouracil and cisplatin (5-fluorouracil at 800 mg/m^2^, days 1–5; cisplatin at 80 mg/m^2^, day 1 of a 5 day cycle). One week after completing the first cycle of chemotherapy, contrast-enhanced computed tomography (CT) was performed to determine the efficacy of treatment. This incidentally revealed ascending aortic thrombus that had not been present 1 month before starting chemotherapy. No thrombus in other vessels or embolism to other organs was observed. Blood tests showed no clotting abnormalities: fibrinogen, 340 mg/dL; prothrombin time/international normalized ratio, 0.95; activated partial thromboplastin time, 23.7 s; D-dimer, 0.6 μg/mL; antithrombin III, 128%; and protein C, 62%. Before chemotherapy, only prothrombin time/international normalized ratio and activated partial thromboplastin time were checked, and both were normal (0.92, and 25.6 s, respectively). The patient was immediately referred to our department. As CT showed a floating, 2 cm thrombus (Fig. 1), we decided to perform emergency surgery because of the high risk of embolism. The cross-section of the ascending aortic diameter measured 33 × 33 mm, with no calcified lesions. The surgery began within 2 h of the referral to our department.

**Fig. 2:**
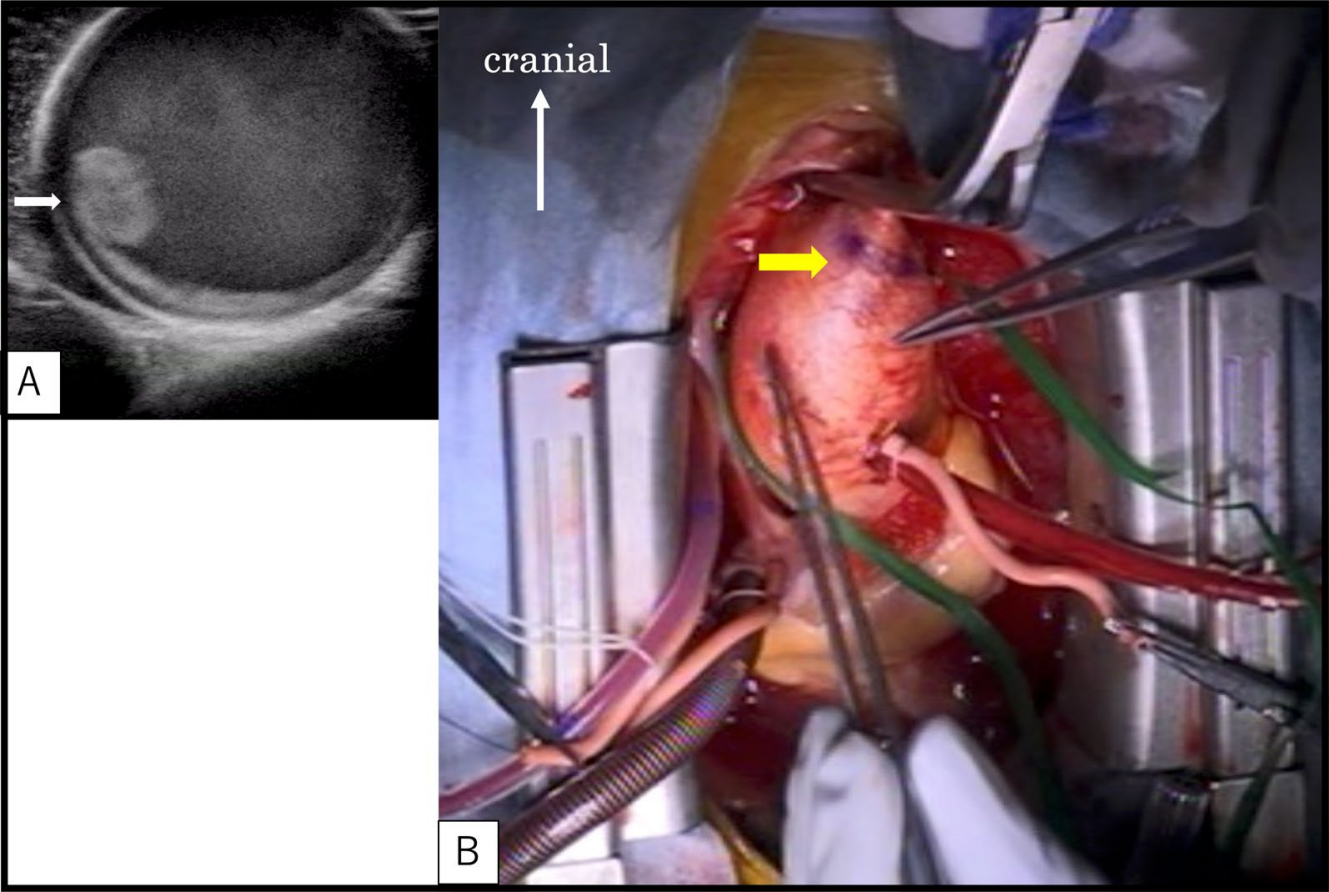
Epiaortic echography confirms the location of the thrombus (white arrow) in the ascending aorta (**A**). We marked the position on the most cephalad side of the floating thrombus (yellow arrow) and determined the locations for aortic cross-clamping and aortotomy (**B**)

**Fig. 3:**
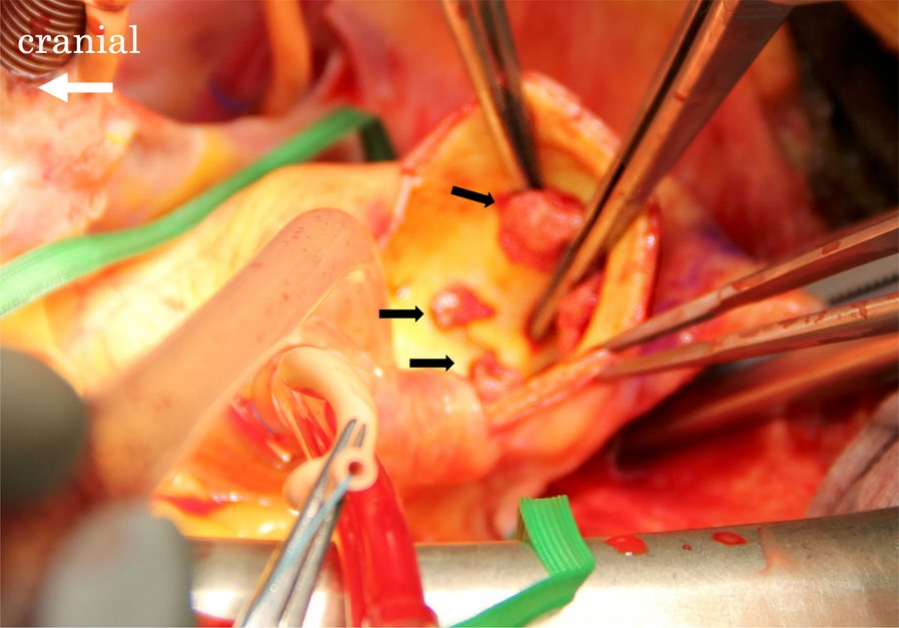
Intraoperative pictures of the aortic intima show three thrombi (black arrows) and only mild atherosclerotic changes

Median sternotomy was performed and the ascending aorta was exposed. Epiaortic echography confirmed the location of one large thrombus and two smaller thrombi in the ascending aorta (Fig. 2). Cardiopulmonary bypass was established by cannulation of the right femoral artery and right atrium, allowing the ascending aorta to be clamped cephalad to the thrombus. Echography showed sufficient space for cannulation proximal to the thrombus, so we decided to cannulate the aortic root for antegrade cardioplegic infusion. To prevent the thrombus from floating away as a result of the change in blood flow, the aorta was immediately clamped as soon as cardiopulmonary bypass was started. After aortic cross-clamping and aortotomy, three thrombi were identified as per echography. The aortic intima showed only mild atherosclerotic changes (Fig. 3).

One of these thrombi was excised by Metzenbaum scissors and the others were easily removed by forceps. Histological analysis of thrombi revealed all were blood clots, with no neoplastic lesions.

The patient was started on low-molecular-weight heparin, warfarin, and aspirin on postoperative day 1. Once the effects of the warfarin were sufficient, heparin was terminated. Postoperative contrast-enhanced CT confirmed no recurrence of thrombus, and he was discharged on postoperative day 16. One month after thrombectomy, the patient underwent surgery for the esophageal cancer. No recurrence of cancer or thrombus was evident as of 6 months after this second surgery, so warfarin was terminated. As of 1 year after the cancer surgery, the patient remains on aspirin only and no recurrences of either cancer or thrombus have been identified.

## Discussion

The risk of thrombosis is known to be increased in patients with carcinoma who receive chemotherapy with cisplatin [1, 2]. However, venous thrombi are more common than arterial ones, and intra-aortic thrombi are rare [3]. The cause has been hypothesized to be endothelial damage, elevation of plasma levels of von Willebrand factor [4], drug-induced reduction of left ventricular function, or hypomagnesemia, although exact causes have yet to be confirmed [2]. Acute aortic thrombosis used to be recognized by embolism to other organs in many cases [5]. Still, in recent years, several reports have described asymptomatic aortic thrombi, partly due to increases in the use of neoadjuvant chemotherapies and associated CT to determine their efficacies [3, 6]. Aortic location, type of chemotherapy, type of cancer, and reason for diagnosis obtained from recent reports of acute aortic thrombosis during chemotherapy are shown in Table 1 [1–3, 7–9]. All these patients were treated with cisplatin-based chemotherapy, and diagnoses were made by re-staging CT or following embolism to some organ. Similar to the reports of no embolism, our patient also received chemotherapy, including cisplatin, and fortunately did not develop embolism before re-staging CT revealed acute aortic thrombosis. Table 1 summarizes the details of the main reports from around the world. Thrombosis has been reported to develop most commonly in the abdominal aorta to lower extremity arteries, although thrombosis of the ascending aorta has also been reported in recent years.

**Table 1:** Recent reports describing acute aortic thrombus with chemotherapy

References	Aortic location	Chemotherapy	Cancer	Diagnosis
Fernandes DD et al. [2]	Abdominal aorta, right common iliac artery	Cisplatin, 5-fluorouracil, folinic acid	Rectosigmoid cancer	Re-staging CT
As above	Abdominal aorta, right common iliac artery	Cisplatin, etoposide	Lung cancer	Vomiting
As above	Abdominal aorta, left common iliac artery	Cisplatin, vinorelbine	Lung cancer	Re-staging CT
As above	Abdominal aorta	Cisplatin, vinorelbine	Lung cancer	Re-staging CT
Hahn SJ et al. [1]	Ascending aorta	Cisplatin, etoposide	Lung cancer	Dyspnea, acute renal infarction
Ito S et al. [3]	Thoracic and abdominal aorta, infrarenal abdominal aorta	Cisplatin, S-1	Gastric cancer	Re-staging CT
Sato C et al. [7]	Thoracic artery to abdominal aorta	Cisplatin, vinorelbine	Lung cancer	Right lower leg ischemia
Yagyu T et al. [8]	Ascending aorta	Cisplatin	Gastric cancer	Re-staging CT
Ochiai Y et al. [9]	Ascending aorta	Cisplatin, etoposide	Lung cancer	Brain infarction

Various treatment strategies are available, but the major distinction is between conservative treatment with anticoagulation and surgical treatment. Anticoagulant therapy was selected when the thrombus was located in the thoracoabdominal aorta, and the surgical treatment was too invasive, or when the patient had cancer and was unsuitable for surgery due to poor general condition. Although reports have described thrombus disappearance with anticoagulation [6], the prognosis is poor in cases of embolism with conservative treatment [10, 11]. Surgical treatment includes thrombectomy only or replacement of the aortic artery. In our case, replacement of the ascending aorta was deemed unnecessary because the thrombi were easily removed and only mild atherosclerotic changes were seen in the aorta. If the diameter of the ascending aorta was more than 55 mm or strong atherosclerotic changes were evident, the patient would have opted for vascular prosthesis replacement would have been recommended, considering the possibility that aneurysm formation or atherosclerosis could be the cause of the thrombus. In the present case, we decided on emergency thrombectomy because we anticipated the floating thrombus to move, and the risk of embolism, including stroke, was thus considered very high [12], and the patient was relatively young and in good condition. In fact, intraoperative findings suggested that the patient was extremely lucky the thrombus had not detached and embolized to another organ.

Distal embolization must be prevented if surgical treatment is chosen. We confirmed the location of thrombus in the ascending aorta by epiaortic echography and planned a treatment strategy. Since the thrombus was located distally in the ascending aorta, femoral artery cannulation allowed for cross-clamping cephalad to the thrombus. To prevent embolism due to the change in blood flow, blood perfusion flow was slowly increased and the aorta was clamped almost as soon as cardiopulmonary bypass started. The ascending aorta showed enough space proximal to the thrombus, so we opted for antegrade cardioplegic infusion, but if space had been unavailable or closer to the thrombus, we would have considered retrograde or selective antegrade cardioplegic infusion.

While various reports have described postoperative anticoagulation, clear evidence on what specific anticoagulant to use remains lacking, so we chose warfarin and aspirin. Any kind of malignancy is known to represent a risk factor for thrombosis [13], so we decided to discontinue warfarin only after first confirming that the chemotherapy had been completed and the cancer had not recurred. Six months after the end of warfarin, the patient remains on aspirin alone, with no recurrence of thrombosis.

## Conclusion

We describe a case of acute aortic thrombosis at the ascending aorta after cisplatin-based chemotherapy, and treated by aortic thrombectomy. The treatment strategy should depend on thrombus location and the condition of the patient, but surgical treatment should be considered where possible to achieve better prognosis.

## Abbreviation

CT: Computed tomography
